# Malaria Policy Advisory Committee to the WHO: conclusions and recommendations of seventh biannual meeting (March 2015)

**DOI:** 10.1186/s12936-015-0787-z

**Published:** 2015-08-05

**Authors:** WHO Malaria Policy Advisory Committee and Secretariat

**Affiliations:** Global Malaria Programme, World Health Organization, 20 Avenue Appia, 1211 Geneva 27, Switzerland

**Keywords:** WHO, Malaria, Policy-making, Mosquito control, Drug resistance, Surveillance, Elimination, *Plasmodium falciparum*, *Plasmodium vivax*

## Abstract

The Malaria Policy Advisory Committee to the World Health Organization held its seventh meeting in Geneva, Switzerland from 5 to 7 March 2015. This article provides a summary of the discussions, conclusions and meeting recommendations. Meeting sessions included: an update on the Greater Mekong Subregion elimination strategy; an update on the RTS,S vaccine; G6PD testing to support the safe use of anti-relapse therapy for *Plasmodium vivax*; update from the Vector Control Advisory Group; newly proposed evidence reviews or consultations on malaria terminology, malaria in pregnancy, and the feasibility of eradication; as well as updates from the World Health Organization Global Malaria Programme regarding their strategy update and policy setting processes. Policy statements, position statements, and guidelines that arise from the Malaria Policy Advisory Committee meeting conclusions and recommendations will be formally issued and disseminated to World Health Organization Member States by the World Health Organization Global Malaria Programme.

## Background

The Malaria Policy Advisory Committee (MPAC) to the WHO held its seventh biannual meeting from 5 to 7 March 2015 in Geneva, Switzerland, following its meetings in February and September 2012, March and September 2013, and March and September 2014 [1–6]. This article provides a summary of the discussions, conclusions and recommendations from that meeting^a^ as part of the *Malaria Journal* thematic series “WHO global malaria recommendations” [7].

The following sections of this article provide details and references for the meeting sessions on: an update on the Greater Mekong Subregion elimination strategy; an update on the RTS,S vaccine; G6PD testing to support the safe use of anti-relapse therapy for *Plasmodium vivax*; update from the Vector Control Advisory Group; newly proposed evidence reviews or consultations on malaria terminology, malaria in pregnancy, and the feasibility of eradication; as well as updates from the WHO Global Malaria Programme (WHO-GMP) regarding their strategy update and policy setting process.

The MPAC discussion and recommendations related to these topics, which took place partially in closed session, are also included. MPAC decisions are reached by consensus [8]. The next meeting of the MPAC will be 16–18 September 2015 [9].

### Report from the WHO Global Malaria Programme

Following a welcome by the chair of MPAC, the Director of WHO-GMP gave an overview of the key findings of the *World Malaria Report 2014* [10]. He provided an update about WHO-GMP’s activities over the past 6 months and the key programme priorities being pursued in WHO regions [11]. Over this period, WHO-GMP issued a number of new technical documents, including guidance on temporary malaria control measures in Ebola-affected countries [12].

Following the update from the Director of WHO-GMP, the Executive Director of the Roll Back Malaria (RBM) Partnership gave an update about the process for finalizing RBM’s *Action and Investment to Defeat Malaria (2016*–*2030)* [13], which will be a companion document to the new *WHO Global Technical Strategy for Malaria (2016*–*2030)* [14]. Development of the two documents has been closely coordinated through seven regional consultations, and the documents have the same goals, milestones and targets. The RBM Executive Director explained that in addition to the regional consultations, the draft RBM document has been reviewed in 12 national consultations, and was shaped through over 120 interviews with key informants. She summarized the seven key priorities established in the draft document, which should drive future efforts to strengthen political commitment, financial resources and the enabling environment for malaria efforts [15].

MPAC welcomed plans by WHO-GMP and RBM to work with countries on translating the new global guidance documents into action at the national level, and highlighted the importance of providing technical support to ensure that national malaria plans are updated and implemented in a timely manner.

### Malaria elimination in the Greater Mekong Subregion

The WHO Regional Malaria Advisors from the South East Asian and Western Pacific Regional Offices jointly presented an update on the Greater Mekong Subregion (GMS) Malaria Elimination Strategy (following the recommendation by MPAC at its previous meeting in September 2014 [6] to adopt a *Plasmodium falciparum* elimination goal in the GMS by 2030 in order to contain multiple foci of artemisinin resistance). The strategy has been drafted under the leader-ship of the WHO Regional Hub for the Emergency Response to Artemisinin Resistance (ERAR), the two Regional Offices concerned (SEARO and WPRO) and WHO-GMP. The update covered: (a) the strategy development process; (b) the proposed goals and targets; and (c) the recommended strategic directions to scale up efforts in order to achieve malaria elimination of all parasite species by 2030. WHO also presented options for a governance structure that could drive efforts forward in a more effective way [16].

Overall, MPAC was supportive of the draft strategy and agreed with the proposed regional and country priorities. The committee noted, however, that the urgency of the response should be emphasized more strongly, consideration should be given to accelerating the timelines, and there should be a more pronounced mention of the need for national political commitment at the highest level. MPAC also discussed the importance of multi-sectoral engagement, the need to clarify the role of “governance” and “management”, and the need to position countries as the main drivers of this effort. The committee also raised the idea of creating an independent monitoring board to assess progress.

After extensive discussion, MPAC members decided to share a written response with the strategy drafting committee, and send a communication to the WHO Director-General highlighting the need to treat this issue as an urgent public health priority with global implications that requires commensurate support and commitment from both WHO leadership and development partners.

### Update on the latest RTS,S vaccine results and analysis

The Joint Technical Expert Group (JTEG) on the RTS,S vaccine presented the latest RTS,S trial results. Considerations about the preparation of a potential WHO policy recommendation, and the preliminary data on the cost-effectiveness and potential impact of RTS,S were also presented. These presentations and the discussions that followed were closed to the public due to the confidential (i.e. unpublished) nature of the results at the time of the MPAC meeting. In addition, the discussions were closed to MPAC members who had declared a conflict of interest due to involvement in the RTS,S Phase 3 trial.

MPAC will review the available information on RTS,S at its next meeting in September 2015. A final decision on a potential WHO policy recommendation will be made at a joint meeting of MPAC and the Strategic Advisory Group of Experts (SAGE)—which is the WHO advisory body for vaccines—in October 2015, if a positive scientific opinion from the European Medicines Agency under article 58 has been given by then.

### Recommendations on testing for glucose-6-phosphate dehydrogenase (G6PD) deficiency to promote safe use of primaquine anti-relapse therapy

An Evidence Review Group (ERG) convened to review new technologies and devices for testing glucose-6-phosphate dehydrogenase (G6PD) deficiency at the point of care presented MPAC with the results from their 8–9 October 2014 meeting [17]. In brief, episodes of *P. vivax* malaria, including relapses, which have been long considered relatively benign, are an important cause of morbidity and mortality in endemic areas. The relapses due to untreated *P. vivax* liver stage infection also make a significant contribution to transmission. Primaquine, the only medicine available for radical cure of *P. vivax* and *Plasmodium ovale* malaria, can induce dose-dependent acute haemolytic anaemia (AHA) in G6PD deficient patients. The severity of the AHA is variable, but daily primaquine anti-relapse therapy can induce potentially life-threatening haemolysis of varying degrees in patients with all known G6PD genetic variants that cause deficiency [18].

Following discussion of the ERG review, MPAC concluded that its recommendations relating to the treatment of *P. vivax* malaria were in line with the soon-to-be-released new edition of the WHO malaria treatment guidelines [19]; therefore the recommendations from this MPAC meeting focus on the use of point-of-care qualitative tests for G6PD deficiency, as follows:

1. G6PD status should be ascertained before the administration of daily primaquine therapy for 14 days to prevent relapses in patients with confirmed *P. vivax* or *P. ovale* malaria.
2. If patients’ G6PD status is not generally available, then G6PD qualitative point-of-care tests should be introduced in *P. vivax* endemic areas to identify G6PD non-deficient patients prior to primaquine administration. Such tests should be at least 95% sensitive for G6PD enzyme activity levels below 30% of normal as identified by spectrophotometry or equivalent quantitative tests. They should be stable at 30–40°C.
3. The introduction of point-of-care G6PD tests should be accompanied by quality assurance, training, supervision, and behaviour change communication as well as monitoring of the feasibility, acceptability and ease of use of these tests. Lessons from initial small-scale deployment should guide decisions on expansion of G6PD diagnostic services across the health care system.
4. The indications and contraindications for primaquine treatment in males and females with G6PD deficiency should follow the recommendations given in the WHO Guidelines for the Treatment of Malaria. Third edition (in press at the time of the meeting).

### Update from the WHO Vector Control Advisory Group

The WHO Vector Control Advisory Group (VCAG) gave a presentation about their November 2014 meeting [20], and briefed MPAC about a recent VCAG recommendation concerning a specific long-lasting insecticidal net (LLIN) product that contains both a pyrethroid insecticide and a synergist. This product is undergoing additional field evaluations and evidence review. VCAG stressed that there is still an urgent need to develop combination LLINs that incorporate multiple insecticides.

MPAC acknowledged the important progress that is being made in product development for malaria vector control and called for continued investments and innovation by industry and others in the private sector. The committee underlined the urgent need for the development and deployment of new insecticides and new tools to prevent and manage insecticide resistance and residual transmission. It also expressed strong support and appreciation for the work done by VCAG. It recommended that WHO-GMP—through the Vector Control TEG—review available data comprehensively and develop recommendations accordingly.

### Malaria terminology and proposed ERG on malaria in pregnancy

WHO-GMP briefed MPAC about plans to review technical terminology for malaria [21]. The last comprehensive review, which included over 400 malaria terms, was undertaken more than 50 years ago [22]. A desk-review process and a drafting committee have been established to take this forward, and the draft document will be shared with MPAC at its next meeting in September 2015. Given the breadth of this exercise, the committee will be reviewing terminology in phases. It will start with those that are most relevant to malaria elimination and eradication, then move on to terms that have programmatic relevance and those that may have conflicting definitions. MPAC welcomed this initiative and some members suggested that WHO-GMP considers turning this into a continuous process for reviewing and updating terminology.

WHO-GMP also briefed MPAC on plans for convening an ERG on malaria in pregnancy [23]. The ERG will review emerging evidence on the efficacy, safety, feasibility, acceptability and cost-effectiveness of using intermittent screening and treatment of malaria in pregnancy (ISTp) to prevent the consequences of malaria in pregnancy [24, 25]. The scope of this ERG will cover: (a) an assessment of whether ISTp should be considered a potential alternative strategy to intermittent preventive treatment of malaria in pregnancy (IPTp) with sulfadoxine-pyrimethamine (SP) in areas with low malaria transmission or high SP resistance; (b) a review of the impact of SP resistance, transmission intensity and threshold maps for potential implementation; and (c) consideration of the safety of using artemisinin derivatives in the first trimester of pregnancy on the basis of emerging evidence. The MPAC welcomed the plan and suggested that toxicology expertise be included in the evidence review process.

### Consultation on the feasibility of malaria eradication

WHO-GMP informed MPAC about its plans to convene leading malaria experts for a review of the feasibility of malaria eradication in the context of increasing interest in this topic within the malaria community. The draft global technical strategy [14] sets out a vision of a “world free of malaria”, and WHO-GMP needs to take an official position on how and under what timeline malaria eradication could be achieved. WHO-GMP would like MPAC to be engaged in a broad technical consultation on the feasibility of eradication, underpinned by a rigorous scientific review, which together will lead to the development of a robust technical document on how the eradication agenda should be taken forward. The document would include a comprehensive research agenda for eradication, and an analysis of options for moving towards that goal.

MPAC agreed to support WHO-GMP in this effort and looks forward to receiving a concrete proposal on how the process will be set up. The committee suggested that WHO-GMP consider using a scenario-based approach i.e. of different pathways to malaria eradication based on various epidemiological profiles, as this would provide a good analytical framework while at the same time helping to make the investment case for malaria in the post-2015 period. It was suggested that WHO-GMP should look at how the various 2030 goals and targets impact on each other, and that this assessment includes an analysis of social, political, economic, environmental and other determinants of epidemiological change.

### WHO-GMP strategy update and policy-setting

The WHO-GMP Director provided an update on the current strategy refresh process within WHO-GMP [26, 27]. The aim of this process is to re-assess and update the department’s strategic priorities in line with the goals and targets of the new *WHO Global Technical Strategy for Malaria (2016*–*2030)* [14]. Following extensive internal consultation, the process will lead to the creation of an updated team structure that is fully aligned with WHO-GMP’s new strategic priorities. WHO-GMP acknowledged that the implementation of the new strategy will require strengthened human and financial resources across the WHO global malaria team. MPAC welcomed the initiative and several members highlighted the importance of strengthening surveillance systems and improving data analysis—on both the disease burden and on interventions—in order to maximize the effectiveness of malaria responses at the national, regional, and global level.

WHO-GMP proposed a streamlining of policy-setting, so that MPAC would not be asked to validate all guidance, but focus on the key strategic and technical questions on which WHO-GMP needs assistance [28, 29]. Concretely, the agenda would be determined from a running list of priority topics kept and reviewed on a monthly basis by WHO-GMP. In general, the reports and recommendations from TEGs and ERGs should be for information, unless they have significant impact, are controversial, or are thought by WHO-GMP to require MPAC advice. MPAC welcomed the proposal by WHO-GMP and recommended that the same rules apply regarding the participation of MPAC members in TEGs and ERGs. MPAC also asked that they continue to receive all TEG and ERG reports.

WHO-GMP further informed MPAC that it would standardize and improve the materials and dissemination of policy recommendations, policy briefs and other guidance to inform national programmes and other stakeholders. This effort will culminate in the consolidation of a “global handbook” to serve as a user-friendly compendium of WHO malaria programme guidance. MPAC members welcomed this development.

## Discussion

The wording for recommendations were finalized by MPAC during their closed session and, in some cases, via email following the meeting; conclusions have been included in the summaries of the meeting sessions above, and links to the full set of meeting documents from the open sessions are provided as references.

As has been done following previous meetings, policy recommendations in line with MPAC suggestions will be issued formally and disseminated to WHO Member States by WHO-GMP and the WHO Regional Offices. Conclusions and recommendations from MPAC meetings are published in the *Malaria Journal* as part of this series.

On-going engagement with and attendance by interested stakeholders at MPAC meetings continues to be strong, although it was noted that more can be done to publicize open registration, especially to encourage attendance by research and development organizations who might not otherwise be aware that their presence as observers, as with all stakeholders who observe the MPAC meetings, is most welcome.

## Conclusion

The meeting feedback received from MPAC members, participants and observers [30] was generally positive. WHO-GMP and MPAC continue to welcome feedback, support, and suggestions for improvement of MPAC meetings from the global malaria community via the WHO-GMP website [9]. The next meeting of the MPAC will take place from 16 to 18 September 2015 in Geneva, Switzerland. Further information including the agenda and registration details will be made available in August 2015 on the MPAC page of the WHO-GMP website, although questions are welcome at any time [9].

## Endnotes

^a^The complete set of all MPAC March 2015 meeting-related documents including background papers, presentations, and member declarations of interest can be found online at http://www.who.int/malaria/mpac/mar2015/en/.

## Abbreviations

AHA: acute haemolytic anaemia
ERAR: WHO emergency response to artemisinin resistance
ERG: Evidence Review Group
G6PD: glucose-6-phosphate dehydrogenase
GMS: Greater Mekong Subregion
IPTp: intermittent preventive treatment of malaria in pregnancy
ISTp: intermittent screening and treatment of malaria in pregnancy
LLIN: long-lasting insecticide treated nets
MDA: Mass Drug Administration
MPAC: Malaria Policy Advisory Committee
RBM: roll back malaria
SAGE: Strategic Advisory Group of Experts
SP: sulfadoxine-pyrimethamine
TEG: Technical Expert Group
VCAG: Vector Control Advisory Group
WHO-GMP: World Health Organization Global Malaria Programme
WHOPES: WHO Pesticide Evaluation Scheme
